# Enhancement of the Functional Properties of Mead Aged with Oak (*Quercus*) Chips at Different Toasting Levels

**DOI:** 10.3390/molecules28010056

**Published:** 2022-12-21

**Authors:** Juciane Prois Fortes, Fernanda Wouters Franco, Julia Baranzelli, Gustavo Andrade Ugalde, Cristiano Augusto Ballus, Eliseu Rodrigues, Márcio Antônio Mazutti, Sabrina Somacal, Claudia Kaehler Sautter

**Affiliations:** 1 Graduate Program on Food Science and Technology, Center of Rural Sciences, Federal University of Santa Maria, Santa Maria, RS 97105-900, Brazil; 2 Integrated Centre for Laboratory Analysis Development (NIDAL), Department of Food Technology and Science, Center of Rural Sciences, Federal University of Santa Maria, Santa Maria, RS 97105-900, Brazil; 3 Graduate Program on Pharmaceutical Sciences, Center of Health Sciences, Federal University of Santa Maria, Santa Maria, RS 97105-900, Brazil; 4 Department of Food Science, Federal University of Rio Grande do Sul, Porto Alegre, RS 91501-970, Brazil; 5 Department of Chemical Engineering, Federal University of Santa Maria, Santa Maria, RS 97105-900, Brazil

**Keywords:** polyphenols, honey, functional beverage, characterization, alcoholic beverage, fermented beverage, phenolic compounds, antioxidant, functional foods, beneficial effects

## Abstract

Consumers increasingly prefer and seek functional beverages, which, given their characteristics, provide important bioactive compounds that help prevent and treat chronic diseases. Mead is a traditional fermented alcoholic beverage made from honey solution. The aging process of mead with oak chips is innovative and bestows functional characteristics to this beverage. Thus, in this study, we sought to develop and characterize a novel functional beverage by combining the health benefits of honey with the traditional aging process of alcoholic beverages in wood. Phenolic compounds, flavonoids, and antioxidant capacity were analyzed in mead using oak chips at different toasting levels and aged for 360 days. LC-ESI-QTOF-MS/MS was used to analyze the chemical profile of different meads. Over time, the aging process with oak chips showed a higher total phenolic and flavonoid content and antioxidant capacity. Eighteen compounds belonging to the classes of organic acids, phenolic acids, flavonoids, and tannins were identified in meads after 360 days. Our findings revealed that the addition of oak chips during aging contributed to *p*-coumaric, ellagic, abscisic, and chlorogenic acids, and naringenin, vanillin, and tiliroside significantly impacted the functional quality of mead.

## 1. Introduction

The increased prevalence of noncommunicable diseases in recent years has made consumers increasingly aware of healthy and natural diets [1]. Consequently, the food and beverages industry faces new challenges in designing functional foods. Among the different types of functional foods, beverages are the most acceptable due to logistic facilities, their distribution, and the ease of incorporating bioactive compounds as functional ingredients [2]. Furthermore, functional beverages have gained more market shares over the last decade [3].

Recently, wine and beer, two of the most popular alcoholic beverages, have been identified as functional beverages, and the benefits of their moderate consumption have been widely supported by the scientific community [4]. The main source of the beneficial potential of consuming these beverages is phenolic compounds. After consuming phenolic-compound-rich foods, such as functional beverages, the colon is the leading site of microbial fermentation. Intestinal microbiota transforms phenolic compounds into phenolic acids or lactone structures, which produce metabolites with biological and antioxidant activity, and evidence suggests that these metabolites have health benefits for humans [3,5,6].

Mead or honey wine is a beverage traditionally produced by diluting honey in water and yeast and may present some variations through the addition of fruit or fruit juice, herbs, or spices [7]. Fermentation and maturation/aging are the two most time-consuming processes in mead production, often lasting a few days to months [8]. The final composition of the mead will depend on the type of honey used, the ingredients added, and the fermentation and storage conditions [9]. Mead has been produced since ancient times, especially in Eastern European countries; however, it is currently not as popular as other alcoholic beverages, highlighting the need for further research on this beverage and its potential functionality [10]. 

Honey is widely known for its health-promoting biological characteristics such as its anti-inflammatory, antiviral, antifungal, and antitumor properties [11]. Among the bioactive descriptors in honey, phenolic compounds, organic acids, carbohydrates, amino acids, proteins, minerals, vitamins, and lipids stand out [11,12], which directly influence the chemical and sensory characteristics of the mead [8,9,12]. Nevertheless, the composition of honey is quite variable and relies on the floral source and seasonal and environmental conditions, in addition to the processing and storage techniques used [9,11]. 

The aging process of alcoholic beverages in wood provides various changes in the composition and concentration of compounds in beverages [13,14]. Such modifications may be noticeable by changes in the beverages’ taste, color, and aroma. In addition to sensory changes, many phenolic compounds are acquired or elevated during maturation [13,15]. Martínez et al. (2008) tested different quenching methods on the chemical composition of American (*Quercus alba*) and French (*Quercus petraea*) oak, and their findings showed the evolution of ellagitannins, a low molecular weight phenolic compound, and volatile compounds regarding oak species and the tempering method [16]. The natural process in the open air was considered superior to the artificial and mixed drying methods, as it showed greater effectiveness in reducing excess ellagitannins. In addition, the evolution of the aromatic potential of the wood was more positive, reaching higher concentrations of compounds such as volatile phenols, phenolic aldehydes, furanic compounds, and *cis*- and *trans*-methyl-γ-octalactones. The wood toasting process takes place after tempering, where the wood is subjected to temperatures in the range of 150–240 °C for a certain period, according to the desired toasting level. In this step, thermal degradation reactions occur, transforming nonvolatile precursors into active aromatic volatile compounds [17].

Due to technological advances in the maturation/aging area of alcoholic beverages, alternative systems using wooden barrels to carry out this process have emerged. Staves, chips, shavings, and other alternative sources of extraction of compounds from oak or other species of forest wood have become common for the maturation and aging process of alcoholic beverages, generating a new use of wood residues in the cooperage [15,18].

Thus, this study aimed to develop a functional mead with multifloral honey and submit it to the aging process with oak chips (*Quercus*), a wood commonly used for the maturation and aging of alcoholic beverages that can provide bioactive compounds. In addition, we sought to evaluate the phenolic compounds content and antioxidant capacity after fermentation and identify the phytochemical compounds in mead aged with oak chips at two toasting levels. 

## 2. Results and Discussion

Functional beverages can be a valuable component of the human diet given their ability to provide essential hydration and important bioactive compounds for maintaining health and/or contributing to preventing and treating chronic diseases [1,2,5,6]. Mead is a beverage that has been gaining more and more notoriety over time, although there are few studies on it; hence, this study is unprecedented as it presents data from meads aged with oak chips, a process so far only used for other types of alcoholic beverages [13] and which can improve the functionality of this beverage. In this study, the antioxidant capacity, total phenolic and flavonoid content, and characterization of phytochemical compounds of meads subjected to a 360-day aging period with oak chips (*Quercus*) at different toasting levels were determined.

Honey is a source of numerous biologically active compounds, including phenolic and volatile compounds, peptides, proteins, amino acids, enzymes, and minerals, which can be transferred to the mead during production [12]. Among all the substances, our attention was given to the phenolic compounds, which contribute to the mead functional quality. This phytochemical group includes flavonoids, tannins, and phenolic acids, which are natural antioxidants that play significant roles in the human body due to their capability to inhibit free radicals, which may cause cell damage, leading to chronic diseases [2,3,19]. Beverages are considered good dietary sources of phenolic compounds; moreover, the phenolic compounds in beverages are highly bioaccessible because they pass directly into intestinal fluids [5], making this study highly innovative in carrying out the aging process of mead with oak chips and monitoring it over time.

**Figure 1:**
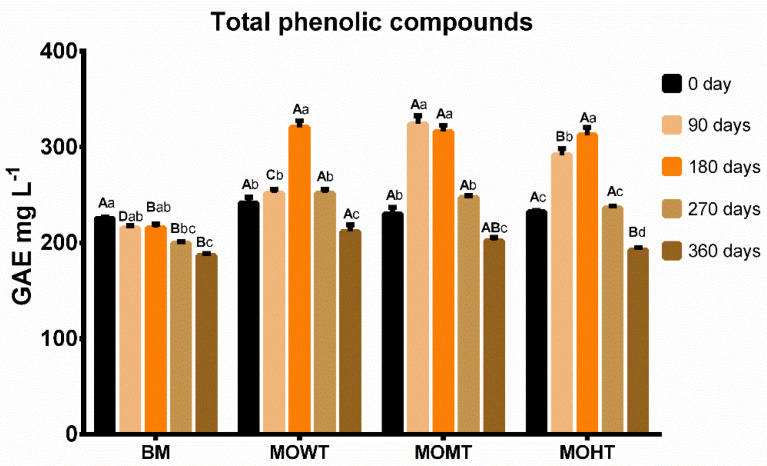
Composition of total phenolics (mg GAE L^−1^) of meads aged with oak (*Quercus*) chips. Data are presented as means ± SEM (*n* = 5). Lowercase letters indicate significant differences over time within the same experimental group (*p* ≤ 0.05). Capital letters indicate significant differences between experimental groups within the same time (*p* ≤ 0.05). GAE: gallic acid equivalent; BM: base mead; MOWT: mead aged with oak chips without toasting; MOMT: mead aged with oak chips at medium toasting; MOHT: mead aged with oak chips at high toasting.

At time zero, all elaborated meads had a similar content of phenolic compounds, which came from the honey used (Figure 1). In the base mead (BM), these values remained constant for up to 180 days and then decreased with advancing maturation time. Meads with chips addition showed higher total phenolic compound levels until the aging period of 180 days. This increase may be related to the extraction of phenolic compounds from oak into mead throughout the maturation process. Canas et al. (2019) observed higher total phenolic compounds in wine spirits aged for 180 days with oak staves [20]. This increase was due to the combination of the thermal degradation of lignin and increased wood permeability during thermal treatment. Mead is still poorly studied, although due to its characteristics similar to young white wines, aging time may have provided oxidative reactions and/or condensation between the mead compounds with some wood molecules, reducing the phenolic compounds after 270 days of aging.

**Figure 2:**
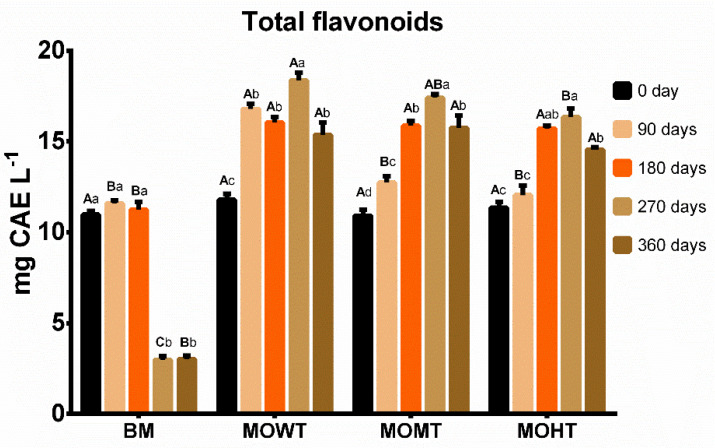
Composition of total flavonoids (mg CAE L^−1^) of meads aged with oak (*Quercus*) chips. Data are presented as means ± SEM (*n* = 5). Lowercase letters indicate significant differences over time within the same experimental group (*p* ≤ 0.05). Capital letters indicate significant differences between experimental groups within the same time (*p* ≤ 0.05). CAE: catechin equivalent; BM: base mead; MOWT: mead aged with oak chips without toasting; MOMT: mead aged with oak chips at medium toasting; MOHT: mead aged with oak chips at high toasting.

**Figure 3:**
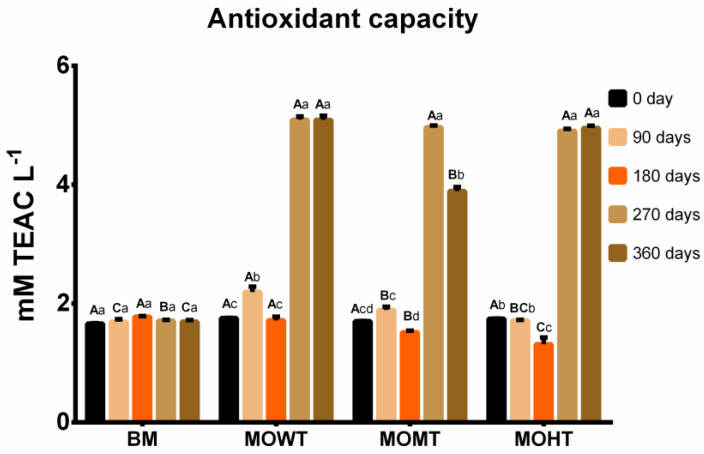
Antioxidant capacity (mM TEAC L^−1^) of meads aged with oak (*Quercus*) chips. Data are presented as means ± SEM (*n* = 5). Lowercase letters indicate significant differences over time within the same experimental group (*p* ≤ 0.05). Capital letters indicate significant differences between experimental groups within the same time (*p* ≤ 0.05). TEAC: Trolox equivalent antioxidant capacity; BM: base mead; MOWT: mead aged with oak chips without toasting; MOMT: mead aged with oak chips at medium toasting; MOHT: mead aged with oak chips at high toasting.

Furthermore, flavonoids gradually increased in meads with oak chips throughout the aging period, with a maximum content at 270 days (Figure 2). We observed that the concentration of this class of phenolic compounds varied from 10.6 mg to 20.5 mg CAE L^−1^. Regardless of the toasting degree, the oak chips increased the mead flavonoids concentrations, which may have contributed to increasing the antioxidant capacity throughout the aging process (Figure 3) since these compounds have high free radical scavenging potential, as described elsewhere [8,19].

The antioxidant capacity of meads depends on the raw material’s chemical composition, the environmental factors that directly affect the honey production process, and the technologies used to process it [8,9]. Apart from technological processes such as fermentation and aging, these beverages’ antioxidant properties and chemical composition are determined by the additives used in their manufacturing [8]. In this investigation, the antioxidant capacity was determined with a 2,2′-azino-bis (3-ethylbenzothiazoline) 6-sulfonic acid (ABTS) assay and varied from 1.31 to 5.06 mM of Trolox equivalent antioxidant capacity per liter (TEAC L^−1^), showing a significant increase after 270 days of aging in meads with the addition of the oak chips (Figure 3). In meads, the antioxidant capacity is related to the presence of phenolic compounds, and the diversity of these compounds is directly linked to the honey used [8]. Hence, meads that only use honey and water in their composition tend to have lower compound diversity.

Nevertheless, meads with the addition of fruit juices or herbal extracts have a wider range of these phytochemicals [12]. The profile of the antioxidant capacity is a concomitant event to the behavior of the flavonoid content over time (Figure 2 and Figure 3), especially at 270 days. A similar behavior was observed in unripened meads and other wood-aged beverages [10,21]. However, one cannot rule out the possibility that other bioactive compounds, in addition to flavonoids, contributed to the antioxidant capacity observed in the final aging period of meads with oak chips in this investigation. Phenolic compounds are found in plants as they come from their secondary metabolism. Bees, by collecting pollen, transfer many of these compounds to the honey; consequently, these phytochemicals will be present in the mead, even in smaller amounts [9,11]. The Brazilian flora is vast, generating honeys with various phenolic compounds, with a predominance of many phenolic acids such as 3,4-dihydroxybenzoic, salicylic, caffeic, chlorogenic, *p*-coumaric, ferulic, gallic, syringic acids, and flavonoids including isorhamnetin, kaempferol, luteolin, naringenin, pinobanksin, quercetin, and rutin [22].

In this study, the phenolic compounds in the meads at the end of 360 days of aging were identified through MS/MS mass spectrometry analysis by combining the chromatographic behavior and collision spectra with compounds already described in the literature. Eighteen compounds belonging to the classes of organic acids, phenolic acids, flavonoids, and tannins were tentatively identified and are listed in Table 1 and in the Supplementary Material (Figure S1).

Among the compounds identified are citric acid, protocatechuic acid, sinapyl alcohol, syringic acid, ethylvanillin, 1-(2-hydroxy-4,6-dimethoxyphenyl)-ethanone, sebacic acid, and quercetin in all samples, which are therefore compounds from the honey used in the beverage’s elaboration. Adding oak chips during aging, regardless of the toasting, contributed to vanillin, *p*-coumaric acid, ellagic acid, abscisic acid, and naringenin. However, chlorogenic acid and tiliroside were only present in the meads aged with oak chips without toasting (MOWT) and meads aged with oak chips at high toasting (MOHT). Various studies have demonstrated that chlorogenic acids are partially bioavailable and potentially beneficial to human health [6]. The antioxidant and anti-inflammatory effects of coffee chlorogenic acids are responsible for, at least to a certain extent, the association between coffee consumption and the lower incidence of various degenerative and nondegenerative diseases, in addition to higher longevity [6]. 2,3-Dihydroxy-1-guaiacylpropanone was only found in the BM, and the 3-hydroxy-3-(3-hydroxyphenyl) propionic acid was found in the BM and mead aged with oak chips without toasting (MOWT).

During the aging process, the transformations of lignin, present in woods such as French oak, are among the most important factors that affect the quality of beverages aged in contact with this material. Lignin is a polymer that undergoes thermal degradation during the manufacturing of barrels or by hydrolysis and ethanollysis during the aging of wines and alcoholic beverages [30]. The lignin macromolecule has ramifications of coniferyl alcohols (guaiacyl compounds) and sinapyl (syringyl compounds). Coniferyl alcohol generates coniferylaldehydes, which are converted into vanillin, while sinapyl alcohol gives rise to sinapaldehyde, which is transformed into syringaldehyde and later oxidized to syringic acid. Other compounds, such as hydrolyzable tannins, present in French oak, are more soluble in hydroalcoholic solutions; its transformation into ellagic acid is very common [31], corroborating the identification of this compound only in meads with the addition of oak chips. 

In the present investigation, meads aged with oak chips showed a higher phenolic compound content and diversity. It is known that these compounds may be related to physicochemical and sensory characteristics in foods [8,12]. Thus, the sensory evaluation of meads is important to know the impact of compounds on sensory aspects, including color, flavor, and astringency. The absence of a sensory evaluation of different meads is one of the limitations of this study. These analyses were scheduled to be carried out in 2020–2021, and the study had already been approved by the institutional ethics committee (CAAE no. 58889316.3.0000.5346). Unfortunately, given the restrictions imposed by the SARS-CoV-2 pandemic, it was impossible to carry out the analyses. Until now, the classic wood-aging method employed in numerous alcoholic beverages has not yet been tested in mead. Hence, despite the absence of a sensory evaluation, this study is pioneering and can contribute to essential elucidations in the field of functional beverages.

Mead is a traditional alcoholic beverage obtained by the fermentation of mead wort and popularly produced at home and in small meaderies. Different types of mead can be distinguished based on the honey-to-water ratio, the addition of spices and/or fruits, and the method of wort preparation [8,10,12]. The consumption of mead has gained popularity given the presence of its natural and high-quality bioactive compounds. As a result, mead production and consumption have remarkably increased during the past years [8]. The regular consumption of foods and beverages rich in bioactive compounds has been associated with a series of beneficial health effects [1,2], although, in the case of mead, in addition to these compounds, this beverage also has a considerable alcohol content (8–18%) [8,10,12]. The excessive and prolonged use of alcoholic beverages is significantly linked to mild symptoms such as fatigue, difficulty walking, fainting, and behavioral changes. In addition, long-term consumption is responsible for serious health problems such as depression, anxiety, impaired cognitive performance, and liver diseases (e.g., alcoholic hepatitis and liver cirrhosis), which is considered one of the main causes of death and functional disability in the world [32,33]. Given the above, the moderate consumption of mead and other alcoholic beverages is suggested.

## 3. Materials and Methods

### 3.1. Analytical Reagents

HPLC-grade methanol used for the mobile phase was obtained from Merck (Darmstadt, Germany). HPLC-grade acetonitrile and formic acid used for the mobile phase were obtained from Sigma-Aldrich (St. Louis, MO, USA). HPLC-grade water was obtained from a Milli-Q system (Millipore, Bedford, MA, USA). ABTS (2,2′-azino-bis(3-ethylbenzothiazoline) 6-sulfonic acid) was obtained from Sigma-Aldrich (St. Louis, MO, USA).

### 3.2. Experimental Design

The experimental design was completely randomized with four treatments and five sampling units for each treatment (Table 2).

### 3.3. Samples Acquirement

To produce the mead we used multifloral honey from an apiary located in Santiago city (29.1991393′ S and 54.8644842′ W, 467 m) in the central region of Rio Grande do Sul, Southern Brazil. It has an area of 2,413 km^2^ of foraging, and the climate is classified as humid subtropical, with an annual average temperature of between 18 and 20 °C and an average annual rainfall of 359 mm. For the aging of mead, we utilized oak chips (*Quercus*) purchased from WE Consultoria (Porto Alegre, Rio Grande do Sul, Brazil). 

The mead was prepared with multifloral honey and water until it reached 21° Brix; then, it was inoculated with 20 hg L^−1^ of the *Saccharomyces baianus* yeast strain and 30 hg L^−1^ of nutrients (Nutristart). Fermentation was carried out in a glass fermenter with a capacity of 30 L with the system maintained under anaerobic conditions through the water seal at a constant temperature of 20 °C, and it was monitored daily by measuring the total soluble solids content and initial and final density. The end of the fermentation process occurred at 27 days with the cessation of carbon dioxide evolution followed by the stabilization of total soluble solids and the stabilization of density. The mead was then stabilized for 15 days at 16 °C. The time between fermentation and the beginning of aging was 45 days. In the last step, the mead was sulfited at 50 ppm and bottled in 300 mL bottles with the addition of 2 g L^−1^ of oak chips (*Quercus*) followed by the aging process. Aliquots were taken every 90 days for up to 360 days to analyze the content of the total phenolic compounds, total flavonoids, and antioxidant capacity. At the end of the 360 days of aging, the phenolic compounds in the different meads were identified.

### 3.4. Total Phenolic Content

The total phenolic compounds in each mead sample were quantified with spectrophotometry through the redox reaction with the Folin–Ciocalteu reagent [34]. The readings of the samples were performed in triplicate of the absorbances in a UV–visible spectrophotometer (FEMTO 600 plus) at a wavelength of 765 nm after they were left to rest for two hours at room temperature. The phenolic compounds content was calculated by interpolating the absorbance of the samples against the calibration curve constituted with a standard of gallic acid (0–80 mg L^−1^), and the results are expressed in milligrams of gallic acid equivalent per liter (mg GAE L^−1^).

### 3.5. Flavonoids Content

Flavonoid compound determination was performed according to the method of Zhishen, Mengcheng, and Jianming (1999) [35]. The absorbance readings were performed in triplicate with a UV–visible spectrophotometer (FEMTO 600 plus) at 550 nm. The flavonoid concentration in the meads was calculated by interpolating the data with the calibration curve constituted with the catechin standard (0–250 mg L^−1^), and the results were expressed in milligrams of catechin equivalent per liter (mg CAE L^−1^).

### 3.6. Antioxidant Capacity Determination

The antioxidant capacity of meads was determined with the ABTS method using the method described by Re et al. (1999) [36]. The absorbance readings of the samples were taken 6 min after the reaction in a UV–visible spectrophotometer (FEMTO^®^ 600 plus) at 750 nm. The ABTS concentration was calculated from a calibration curve using Trolox as the standard (0–0.2 mM TEAC L^−1^). Readings were performed in triplicate, and the results are expressed as mM of Trolox equivalent antioxidant capacity per liter (mM TEAC L ^−1^).

### 3.7. Purification Procedure in SPE C18

Samples were purified prior to performing the LC-ESI-QTOF-MS/MS analysis. Sample purification was performed according to the method described by Rodriguez-Saona and Wrolstad (2001) with adaptations [37,38]. The mead samples (6 mL) were placed in a rotary evaporator (Büchi, Essen, Germany) at 35 °C for five minutes to remove the alcohol present in the sample. Afterward, the sample was loaded into C-18 solid phase extraction (SPE) cartridges (cartridges SPE-C18, Strata C18-E, Phenomenex), previously activated with methanol and conditioned with acidified water (0.1% *v*/*v* formic acid). The polar compounds were washed with two volumes of aqueous formic acid solution (0.1% *v*/*v*). Fewer polar phenolic compounds were eluted with two volumes of ethyl acetate (3 mL). The ethyl acetate fraction was dried on a rotary evaporator and made up to a known volume (1 mL) with acidified methanol (0.1% *v*/*v* formic acid) and acidified water (0.1% *v*/*v* formic acid) (200 + 800 µL). All fractions were analyzed directly as purified fractions in a chromatograph.

### 3.8. Phenolic Compound Identification by LC-ESI-QTOF-MS/MS

The method to identify the phenolic compounds was based on Quatrin et al. (2019) [39]. The liquid chromatography (LC) instrument (Shimadzu, Kyoto, Japan) was connected in series to a DAD detector (SPD-M20A) and a mass spectrometer (MS) with a Quadrupole-Time-of-Flight (QTOF) analyzer and an electrospray ionization source (ESI) (Bruker Daltonics, micrOTOF-Q III, Bremen, Germany). A 20 µL sample was injected into a reversed-phase column (C-18 Hypersil Gold, 5 μm particle size, 150 mm, 4.6 mm; Thermo Fisher Scientific, Waltham, MA, USA). Mobile phase A for this method consisted of ultrapure water with formic acid acidified methanol (95:5:0.1 *v*/*v*); mobile phase B was acetonitrile and formic acid (99.9:0.1 *v*/*v*). The ESI conditions were a capillary voltage of −4500 V (negative), nebulizer gas pressure at 30 psi, dry gas at 11 mL min^−1^, and gas temperature at 310 °C. The MS/MS experiments were performed in a full scan range of 100–1800 *m*/*z* of all fragments formed from 3 major parent ions per second. The LC solutions software (Version 3, Shimadzu, Kyoto, Japan) was used to process the data obtained. The tentative identification of compounds was based on the combined information of elution order, ultraviolet–visible (UV–Vis) spectra, and mass spectrometry fragmentation patterns. These data were compared to literature data and public databases (PubChem, KEGG, MassBank of North America (MoNA), ChemSpider, Phenol-Explorer, and FooDB).

### 3.9. Statistical Analysis

All analytical results were selected for an analysis of variance (ANOVA). The comparison of posthoc mean analysis was performed with a Tukey’s test at 5% error probability using Statistica 9.0 software (StatSoft, Tulsa, OK, USA); the graphs were made using the GraphPad Prism 6.0 software (Dotmatics, San Diego, CA, USA).

## 4. Conclusions

The highly innovative process of using oak chips to improve mead characteristics has not yet been described in the literature. Oak chips increase phenolic compound variability in mead, their flavonoid content, and their antioxidant capacity over storage time. Our findings revealed that mead aged with oak chips as a beverage has more potential for beneficial biological activity due to the higher phenolic compound content than mead without oak chips. Therefore, the use of oak chips in the mead aging process, regardless of toasting levels, improved the functional quality of the beverage. 

## Figures and Tables

**Table 1:** Phytochemical compounds detected in mead produced with multifloral honey aged with oak (*Quercus*) chips.

Tentative Indentification	RT (min)	Molecular Formula	Molecular Weight	Theoretical (*m/z*)	Observed (*m/z*)	Fragmentation Ion (*m/z*)	BM	MOWT	MOMT	MOHT	Reference
Citric acid	2.1	C_6_H_8_O_7_	192.0270	191.0197	191.0368	111.0205	X	X	X	X	[12]
3-Hydroxy-3-(3-hydroxyphenyl) propionic acid	9.0	C_9_H_10_O_4_	182.0579	181.0506	181.0533	121.0420/122.0480	X	X			[23]
2,3-Dihydroxy-1-guaiacylpropanone	9.2	C_10_H_12_O_5_	212.0685	211.0612	211.0789	134.0499/150.0450	X				[23]
Chlorogenic acid	9.2	C_16_H_18_O_9_	354.0951	353.0878	353.0899	191.0574		X		X	[22]
Protocatechuic acid	9.3	C_7_H_6_O_4_	154.0266	153.0193	153.0335	109.0376	X	X	X	X	[12]
Butanedioic acid	10.6	C_8_H_14_O_5_	190.0841	189.0768	189.0791	129.0680/127.0878/99.0934		X	X		[24]
Vanillin	10.7	C_8_H_8_O_3_	152.0475	151.0403	151.0414	108.0181		X	X	X	[12]
Sinapyl alcohol	10.7	C_11_H_14_O_4_	210.0892	209.0819	209.0996	137.0266	X	X	X	X	[25]
Syringic acid	10.8	C_9_H_10_O_5_	198.0528	197.0455	197.0477	111.0182/125.0360/140.0247	X	X	X	X	[22]
Ethylvanillin	11.0	C_9_H_10_O_3_	166.0630	165.0557	165.0583	119.0514/117.0355	X	X	X	X	[12]
*p*-Coumaric	11.1	C_9_H_8_O_3_	164.0473	163.0401	163.0401	119.0518		X	X	X	[10]
1-(2-hydroxy-4,6-dimethoxyphenyl)-ethanone	11.4	C_10_H_12_O_4_	196.0736	195.0663	195.0690	117.0337/134.0387	X	X	X	X	[26]
Ellagic acid	11.6	C_14_H_6_O_8_	302.0063	300.9990	301.0017	229.0170/301.0018		X	X	X	[27]
Abscísic acid	17.1	C_15_H_20_O_4_	264.1362	263.1289	263.1313	136.0543/203.1091		X	X	X	[22]
Sebacic acid	17.5	C_10_H_18_O_4_	202.1205	201.1132	201.1302	183.1170/139.1259	X	X	X	X	[28]
Quercetin	17.7	C_15_H_12_O_5_	302.0427	301.0354	301.0584	151.0175/107.0253/116.0828/121.0426	X	X	X	X	[22]
Naringenin	18.5	C_15_H_10_O_7_	272.0685	271.0612	271.0632	125.0266/197.0639/225.0540/253.0480		X	X	X	[10]
Tiliroside	20.7	C_30_H_26_O_13_	594.1373	593.1301	593.1326	121.0298/209.0480/417.0965		X		X	[29]

BM: base mead (only honey); MOWT: mead aged with oak chips without toast; MOMT: mead aged in oak chips in medium toast; MOHT: mead aged in oak chips in high toast.

**Table 2:** Coding and characterization of treatments used in this study.

Code	Treatment
BM	Base mead—not aged with oak chips
MOWT	Mead aged with oak chips without toasting
MOMT	Mead aged with oak chips at medium toasting (170 °C for 35 min)
MOHT	Mead aged with oak chips at high toasting (200 °C for 45 min)

## Data Availability

The data presented in this study are openly available in the Harvard Dataverse at doi: 10.7910/DVN/MBWA4J.

## Supplementary Materials

The following supporting information can be downloaded at: https://www.mdpi.com/article/10.3390/molecules28010056/s1, Figure S1: Representative chromatogram of mead aged with oak chips.

## Sample Availability

All meads samples are available from the authors.
