# Supplementary material for: Enhancement of the Functional Properties of Mead Aged with Oak (Quercus) Chips at Different Toasting Levels
Source: Molecules. 2022 Dec 21;28(1):56. doi: 10.3390/molecules28010056 (PMC9822390; doi:10.3390/molecules28010056)
Supplement: Supplementary file 1 [file molecules-28-00056-s001.zip › molecules-2065728 Published - Supplementary material.pdf]

Supplementary Material

Enhancement of the functional properties of mead aged with oak (*Quercus*) chips at different toasting levels

Juciane P. Fortes[1,2], Fernanda W. Franco[1,2], Julia Baranzelli[1,2], Gustavo A. Ugalde[3], Cristiano A. Ballus[1], Eliseu Rodrigues[4], Márcio A. Mazutti[5], Sabrina Somacal[1,2,*], Claudia K. Sautter[1,2]

Abstract: Consumers increasingly prefer and seek functional beverages, which, given their characteristics, provide important bioactive compounds that help prevent and treat chronic diseases. Mead is a traditional fermented alcoholic beverage made from honey solution. The aging process of mead with oak chips is innovative and bestows functional characteristics to this beverage. Thus, in this study sought to develop and characterize a novel functional beverage by combining the health benefits of honey with the traditional aging process of alcoholic beverages in wood. Phenolic compounds, flavonoids, and antioxidant capacity were analyzed in mead using oak chips, at different toasting levels, and aged for 360 days. LC-ESI-QTOF-MS/MS was used to analyze the chemical profile of different meads. Over time, the aging process with oak chips showed higher total phenolic and flavonoid content and in antioxidant capacity. Eighteen compounds belonging to the classes of organic acids, phenolic acids, flavonoids, and tannins were identified in meads after 360 days. Our findings revealed that the addition oak chips during aging contributed to *p*-coumaric, ellagic, abscisic, and chlorogenic acids; naringenin, vanillin, and tiliroside significantly impacted the functional quality of mead.

Keywords: polyphenols; honey; functional beverage; characterization; alcoholic beverage; fermented beverage; phenolic compounds; antioxidant; functional foods; beneficial effects

1. Phenolic compound tentative identification

In the analysis of phytochemical compounds in meads by LC-ESI-QTOF-MS/MS it was possible to identify 18 compounds (Figure S1). Tentative identification was performed based on retention time, elution order and MS spectra compared to literature data.

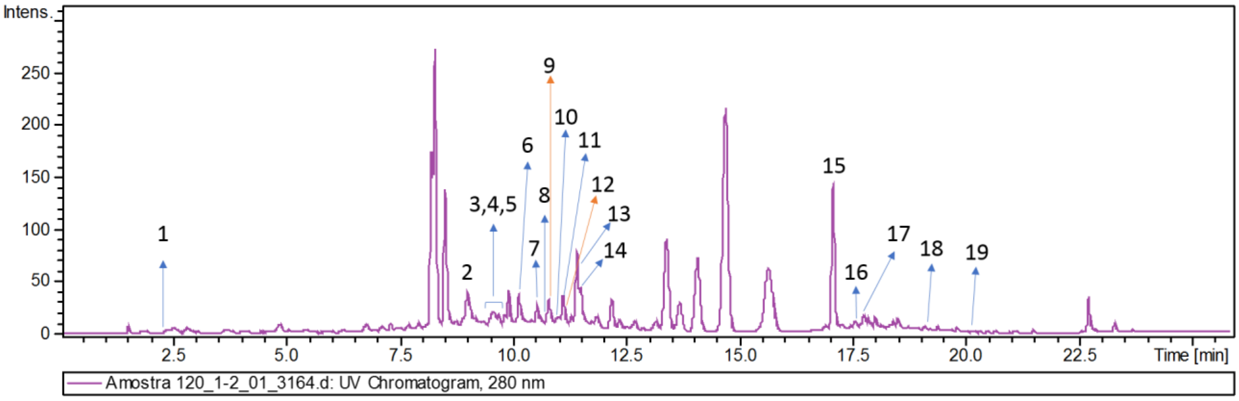

Figure S1 – Representative chromatogram of mead aged with oak chips

Compound 1 (citric acid) was detected with a precursor ion at m/z 191, which produced ion m/z 111.

Compound 2 (3-hydroxy-3-(3-hydroxyphenyl) propionic acid) was tentatively identified by a molecular ion at m/z 181 in negative ionization mode.

2,3-Dihydroxy-1-guaiacylpropanone (compound 3) exhibiting precursor ion at m/z 211 in ESI− mode.

Compound 4 identified as chlorogenic acid presented precursor ion m/z 353 and showed characteristic product ion m/z 191 which corresponds to the deprotonated quinic acid.

Compound 5 (Protocatechuic acid) was detected with a precursor ion at m/z 153.0335 [M-H]- and base peak ion at m/z 109.0376.

Compound 6 (not identified) was detected with a precursor ion at m/z 281.1609 [M-H]-, which produced MS/MS base peak ion at m/z 137.1136; 171.1336, and 189.1480.

Compound 7 butanedioic acid was detected with a precursor ion at m/z 189.0791[M-H]- which produced MS/MS base peak ion at m/z 129.0680; 127.0878 and 99.0934.

Compound (peak 8) was detected with a precursor ion at m/z 151.0414 [M-H]- with base peak ion 108.0181.

Compound 9, sinapyl alcohol was detected with precursor ion at m/z 209.0996 [M-H]- with base peak ion 137.0266.

Compound 10 (syringic acid) was detected with a precursor ion at m/z 197.0477 produced fragment íons 111.0182; 125.0360; 140.0247 m/z.

Compound 11 (ethylvanillin) was detected with a precursor ion 165.0583 and produced fragment ions at m/z 119.0514/117.0355.

Compound 12 was identified as p-Coumaric acid furnished a deprotonated molecule [MH] at 163.0401 m/z and an $[M\text{-}H]^-$ ion at 119.0518 m/z.

Compound 13 was identified as 1-(2-hydroxy-4,6-dimethoxyphenyl)-ethanone with a precursor ion at m/z 195.0690 and produced MS/MS base peak ion at m/z 117.03371 and 134.0387.

Compound 14 (ellagic acid) shows a precursor ion at 301.0017 with produced MS/MS base peak ion at 229.0170 and 301.0018.

Compound 15 (abscisic acid) was trying to identify with a precursor ion at m/z 263.1313 and produced the fragment ion at m/z 136.0543 and 203.1091.

Compound 18 (naringenin) shows a precursor ion at 271.0632 with produced MS/MS base peak ion at m/z 125.0266/197.0639/225.0540/253.0480.

Compound 19, the aglycone quercetin with m/z 301.0584 produced the fragment ion at m/z 151.0175; 107.0253; 116.0828 and 121.0426.
